# Facilitating learning of community-based rehabilitation through problem-based learning in higher education

**DOI:** 10.1186/s12909-019-1868-4

**Published:** 2019-11-21

**Authors:** Eva Yin-han Chung

**Affiliations:** Department of Special Education and Counselling, The Education University of Hong Kong, 10 Lo Ping Road, Tai Po, New Territories Hong Kong

**Keywords:** Community-based rehabilitation, Problem-based learning, Health science education, Pedagogy

## Abstract

**Background:**

The quality of community-based rehabilitation (CBR) personnel is one of the key factors that contributes to the success of CBR programs. Integrating knowledge and practical skills in various stages of the learning process is essential in community-based rehabilitation. Problem-based learning (PBL) is a pedagogical strategy that uses real-world situations as the basis for developing knowledge and problem-solving skills. Through PBL, learners are guided and facilitated in assuming active problem-solving roles in real-world situations. This study developed and tested a framework and a PBL protocol for use in teaching community-based rehabilitation (CBR) in higher education.

**Methods:**

Part I of this study focused on the development of a framework and a protocol for PBL. An initial framework for the development of this protocol was formed based on a review of relevant literature. Concrete guidelines were delineated to describe the application, process, and delivery of teaching and learning. PBL was implemented in three CBR related courses. Students were facilitated to learn CBR in passing various stages of PBL through a self-directed learning process. The cumulative efforts of each group were compiled, recorded, and displayed using e-portfolios. In Part II, the processes and outcomes of using this new learning mode were evaluated using a case study approach to examine the protocol’s efficacy. Focus group interviews, a questionnaire, and a detailed examination of the e-portfolios were administered for evaluation.

**Results:**

One hundred thirty-three students from three CBR related courses were recruited. PBL was regarded as an effective, realistic and practical method that enables critical thinking in CBR. Practicality was addressed by covering context-related materials with the use of real cases or examples. Participants were actively engaged in the learning process and their CBR competence was enhanced.

**Conclusions:**

Through the new protocol, the students were equipped with active learning, critical thinking, and problem-solving skills that should facilitate success in CBR.

## Background

Community-based rehabilitation (CBR) has been implemented in over 100 countries for over three decades [1]. It is a multisectoral approach that works to achieve equal opportunities and social inclusion of people with disabilities and combat the perpetual cycle of poverty and disability. The quality of CBR personnel is one of the key factors that contributes to the success of CBR programs [2]. CBR personnel include grassroots workers, mid-level practitioners, and professionals [3]. These practitioners and professionals have normally completed a degree program in health, rehabilitation, education, or social work, and their primary tasks in a programme consist of coordination, service planning, supervision, staff training, and case management [4]. Other research has largely focused on the education and training of frontline CBR workers [5, 6] . Because CBR has been developing for more than 30 years and has been incorporated into most health-related higher educational programmes, it is essential to find an effective manner through which to teach CBR to future CBR practitioners in higher education settings to ensure they can perform competently in CBR programs.

CBR leaders and practitioners must have a high degree of flexibility and innovative thinking and possess a wide range of management, practice, teaching, and learning skills to work effectively in CBR because these capacities endow them with an ability that transcends medical interpretation to comprehend the needs of the individual in a wider population [7]. CBR educators must provide learning experiences that establish knowledge-seeking behaviour to facilitate the development of reasoning skills and cultural competency critical to CBR [8]. CBR practitioners must develop a passion for community development and a culturally respectful attitude to adopt a person-centred approach that is necessary to work with people with disabilities and their families. However, there is yet no published articles or studies that examine how CBR should be taught in higher education. Most curricula of the university-educated practitioners’ programmes would have been based on a didactic pedagogy which makes the learning of CBR less effective [8].

Innovative teaching methods, workshops, and other small group activities have been identified as useful means for facilitating cultural competency and reasoning skills in CBR practitioners [8]. This study advocates for the use of a problem-based learning approach to teach CBR in higher education. The using of a PBL pedagogy to teach CBR in higher education can be robust but its evidence base is lacking. Transferring knowledge into practice is a particularly prevalent concern among educators who provide training in complex health science–related disciplines; such educators include occupational therapists, physical therapists, nurses, and special education specialists because such disciplines require students to not only understand theories comprehensively but also to apply them flexibly in a dynamic cultural and community context [9]. Conventionally, educators devote considerable amounts of time delivering theoretical information in lectures and seminars through a one-way communication strategy in which the student is a mere passive learner. This approach is not preferred and has been criticized because of its overreliance on one-way learning. Consequently, students tend to experience difficulty when called upon to transfer theoretical knowledge into practical application and exhibit an insufficient capacity to acquire critical knowledge [10].

Problem-based learning (PBL) was developed as a method for transferring theory into practice [11]. It is a learner-centred teaching approach that facilitates learning by encouraging students to apply theories to solve problems in real situations [12, 13]. Its aim is to help students to develop knowledge, problem-solving skills, and the motivation to learn, thus becoming independent learners [14]. PBL was first adopted in the medical school at McMaster University in Canada in the 1960s [15]. In contrast to traditional teacher-centred lecturing in which a large quantity of specific content is taught directly to students but without providing them with an illustrative clinical application contextual grounding, PBL is a student-centred instructional, and curricular approach that seeks to empower students to identify problems and gaps in their knowledge, conduct research, integrate theory into practice, apply skills and knowledge, and develop solutions to solve problems [13]. PBL emphasizes a learner’s ability to reconstruct their experiences and grow, and the role of a teacher is to activate students’ prior knowledge on the foundation of which they can continually build new experiences. This idea serves as one of the fundamental principles on the basis of which PBL is structured. The ultimate educational goals of PBL are to cultivate students who can engage in self-directed and lifelong learning [16]. Learning through problem-solving is arguably more effective than traditional memory-based learning in fostering practical and critical thinking skills. The most crucial skills for CBR practitioners in real-world practice are problem-solving and critical thinking [17]. Problems are the central component and starting point for teaching and learning in PBL pedagogy. In a PBL setting, a problem should be closely related to the students’ future professions and real-world situations and chosen on the basis of the teacher’s prior knowledge of the students’ abilities to apply knowledge and skills to determine potential solutions to problems [18].

PBL has been found to be an effective teaching pedagogy. Strobel and Van Barneveld [19] conducted a meta-analysis comparing PBL to the traditional approach, and the results demonstrated that PBL was associated with relatively more favourable long-term knowledge retention but traditional teaching methods were associated with relatively more favourable short-term recall of knowledge. Achieving long-term retention is more beneficial for students who seek to understand material, and thus PBL is a more favourable approach because it activates prior knowledge and stimulates elaboration and integration of new information with existing knowledge [20–22]. Additionally, students instructed with a PBL-based curriculum outperformed those instructed with a traditional approach in a study in which learning outcomes were related to performance and skill acquisition [19] PBL enables individuals to learn in an authentic context through the use of a real-life scenario as the focal problem [23]. This accords with study results that have demonstrated that students in a PBL curriculum can more adeptly apply learned knowledge to solve clinical problems than students instructed through traditional means [24–26].

This study developed and tested a PBL protocol for the instruction of CBR in higher education. Instruction in CBR must be embedded across the curricula and permeate all aspects of the educational process [8]. This study consisted of the following two parts: (1) the development of a framework to guide the application of PBL in CBR and (2) an evaluation of learning experiences and outcomes. The two parts of the study were designed to, respectively, answer the following research questions.

1. What does a good practice PBL programme design that is conducive to effectively learning CBR look like?
2. Do PBL programmes that incorporate the good practice design effectively enhance students’ learning processes?

## Methods

A case study approach was used in this study. It is a strategy of inquiry used in qualitative research, in which the investigator explores a case or a system through detailed, in-depth data collection involving multiple source of evidence [27]. A case study approach was adopted for an indepth and detailed examination of the new mode of learning including its processes, outcomes, and impact on the future CBR practitioners. In this study, the PBL mode for learning CBR was the focus and the students were the multiple cases for the study.

### Part I: development of a protocol to guide the application of PBL in education of CBR

Part I of this study focused on developing a protocol. An initial framework for the development of such a protocol was formed based on a review of relevant literature. Concrete guidelines were delineated to describe the application, process, and delivery of teaching and learning methods. The developed protocol asserts that PBL is cumulative and the process of learning is iterative.

#### The problem-based learning framework

The initial framework was derived from a review and synthesis of previous research. A systematic analysis of the literature on PBL was performed to identify the core elements and steps of PBL. Two databases, CINALHL and MEDLINE, were searched using keywords including ‘problem-based learning’ and ‘health science’. Articles published from 2008 to 2017 were included. The two reviewers screened and purposefully selected the relevant papers to build a problem-based learning framework. Thematic analysis was used to examine and collate the findings. Any articles describing the essential steps, processes and core elements of PBL were included for further analysis. Among the 118 articles extracted from the databases, 14 were used for thematic analysis.

**Fig. 1:**
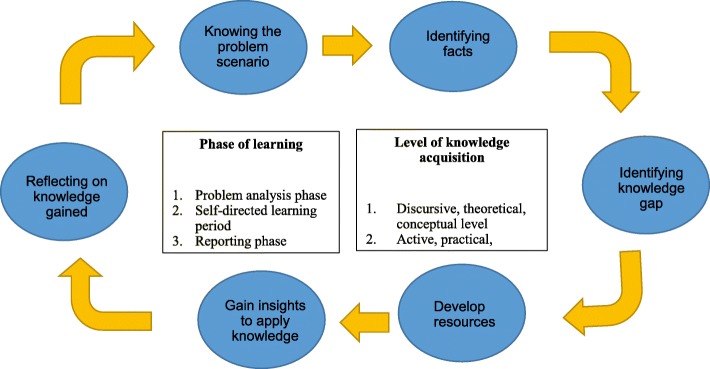
The Problem-based Learning Framework

Thematic analysis was done in six steps [28]. Firstly was familiarization with data. The research team read and reread data, and highlighted the steps, processes and core elements of PBL. The second steps was generating initial codes. Features of interest in the data were coded in a systematic way across the entire data set. The third step was searching for themes. Codes were collated into potential themes. The fourth step was reviewing the themes. The relations of themes and codes regarding PBL were checked and a thematic map of analysis was generated. Thematic map showing the steps of PBL are shown in Table 1. The identified themes related to the steps and procedures of PBL were (1) knowing the problem scenario [29–33]; (2) identifying facts [30–34]; (3) identifying knowledge gaps [29–33, 35]; (4) developing resources [29–33, 35]; (5) gaining insights into applying knowledge [29, 32, 34, 35]; and (6) reflecting on knowledge gain [29–31, 34]. These themes formed the steps of the PBL cycle shown in Fig. 1. A tutor guides the students through the three major phases of learning: (1) problem analysis, (2) self-directed learning and (3) reporting [36]. In the self-directed learning period, students study the issues thoroughly through self-study and group study. The process of knowledge acquisition comprises the following two levels: (1) the discursive, theoretical, conceptual level, and (2) the active, practical, experiential level [37].

**Table 1:** Thematic map on essential steps of PBL

Theme	Codes
Knowing the problem scenario	Presentation of a problem [29]
	Start with a cognitive question [30]
	Knowing the problem [28, 31]
	Define the problem [29]
	Identify the problem [32]
Identifying facts	Collection of information [28, 31]
	Monitor and analyze practice [33]
	Clarify unclear terms and concepts [29]
	Clarify concepts [32]
Identifying knowledge gaps	Establishment of learning objectives [28, 31]
	Generate hypothesis [34]
	Identify objectives [34]
	Engage in learning [34]
	Reexamine and reassess what we have already known [30]
	Formulate learning goals [29, 32]
	Problem analysis and statement formation [32]
Develop resources	Brainstorm [32]
	Prioritize and research through the objectives [34]
	Assimilate knowledge (new and old) [30]
	Read, study, analyze to gain knowledge [30]
	further observation and experiment [30]
	Problem analysis: produce as many ideas a possible [29]
	problem analysis: arrange the ideas systematically and analyze them [29] Summarizing [28, 31]
	Analyze the problem [32]
	independent self-directed study [32]
	discussion [32]
Gain insights to apply knowledge	Inform and apply acquired information to the problem [34]
	apply new learning or improvement [33]
	making inference [30]
	synthesize and apply the new information [29]
Reflecting on knowledge gain	Reflection [28, 31]
	Monitor effects of leaning [33]
	reflective thought [30]

#### The problem-based learning protocol

The PBL protocol was derived from the same review. The elements of PBL were extracted from the literature and analysed (Table 2). The identified themes related to PBL elements were small group learning [31, 34, 38, 39], problem-solving [31, 34, 35], active learning [29, 31, 32, 34], seeing problems in context [34, 35, 40, 41], tutoring [30, 33, 35, 39], writing reflections [34, 35] and using technology [40, 42]. A group work-based, learner-centred, problem-oriented teaching protocol was designed, which consisted of the elements of PBL. It adopted the central values of PBL as guidelines for a professional education teaching method. The protocol could be implemented as a guide to course teaching geared towards enhancing critical thinking in professional practices. A course normally runs over a single 13-weeks semester. The operation on this new mode incorporating the core components of PBL is shown in Table 3. Course content is divided into modules and presented as various checkpoints Table 4.

**Table 2:** Thematic map on core elements of PBL

Theme	Subtheme
Small group learning	work in small group [28]
	group members comprehend the problems and communicate it in the group [35]
	leaders with group-leadership skills [33]
	students placed in small groups to solve problems [37]
	provide students with an opportunity to develop group-based working skills [38]
Problem-solving	encounter the problem-solving situation [28]
	think logically in a organized manner [36]
	use of evidence based strategies [34]
	problems being identified by participants or consulting clinician [33]
Active learning	they need to identify the problems and try to understand it [28]
	they need to identify learning needs [33]
	Investigated a specific clinical problem or task [33]
	inspire and motivate to seek the answer [30]
	Self-study [29]
Problems in context	adopt clinical case scenarios [34]
	apply practice into theories [34]
	use case scenarios [39]
	topics relevant to real context [33]
	Community-focused simulation exercises [40]
	Learning in context [30]
Tutoring	Tutoring-oriented strategies [34]
	intensive tutoring strategies [34]
	use small group tutorials [34]
	use a tutor to facilitate and guide [31, 38]
	complex problem solving tutorial [38]
	Group facilitation [32]
Reflection	Use reflective writing [34]
	reflective on practice problem [33]
Technology	use digital media [39]
	use educational technologies [41]

**Table 3:** The PBL protocol

Week	Knowledge acquisition	The evolving phases	Stages of PBL	Outcome of the learning process for assessment of learning	Embedded PBL elements
1, 5, 9	Conceptual learning	Checkpoint 1/2/3 – Knowing the scenario	Knowing the problem		Seeing the problems in context
2, 6, 10	Active, practical and experiential learning	Checkpoint 1/2/3 – problem analysis and generate hypothesis	Identifying facts		Small group learning
					Tutoring
			Identifying knowledge deficiencies		Problem-solving
					Active learning
					Seeing the problems in context
					Using technology
3, 7, 11	Active, practical and experiential learning	Checkpoint 1/2/3 – self-directed learning	Develop resources		Small group learning
			Gain insights		Tutoring
			Apply new knowledge		Active learning
					Seeing the problems in context
					Problem-solving
					Using technology
4, 8, 12	Active, practical and experiential learning	Checkpoint 1/2/3 - reporting	Reflecting on knowledge gained	Presentation 1/2/3	Small group learning
					Tutoring
					Problem-solving
					Reflection
					Using technology
					Active learning
13	Active, practical and experiential learning	Conclusion and consolidation	Reflecting on knowledge gained	E-portfolio and debate	Problem-solving
					Reflection
					Using technology

**Table 4:** Checkpoints and problem scenarios in the three courses

Sensory and physical disabilities		
Checkpoints		Problems
1	Needs of children with physical disabilities	What are the needs of children with physical disabilities?
		In view of the needs of children with disabilities, how inclusion of children with physical disabilities can be achieved in local community?
2	Activity planning for students with physical disabilities	What if we have to plan for an outdoor activity for a group of school-aged children with physical disabilities?
		How can we arrange such activity to ensure participation of all students with physical disabilities?
3	Barrier free access for students with sensory disabilities	What are the good practice and barriers to free accessibility of persons with visual impairment in a school campus?
		How can we advocate for the rights of people with visual impairment in terms of barrier free access to facilities?
Culturally relevant practice		
1	Beliefs and values. Where am I?	What are the predominating values and cultural norms in Chinese communities?
		What are your reflections related to the above mentioned values and norms?
2	Living with meaning.	With reference to the previous check-point, how the predominating values and cultural norms of local people is affecting the meaning of the occupation among the people in Hong Kong, especially in the areas of daily living, work and leisure?
		How CBR practitioners can help with their clients to define and live out the meaning of occupations with respect to different types of service setting?
3	Life with choices.	What will be the opportunities or challenges of CBR practitioners regarding the increasing emphasis on human right?
		How we can appropriately deal with such challenges.
Enabling occupation - community		
1	Needs assessment using photovoice	Explore the needs of people with disabilities using photovoice.
		How can practitioners work with the people with disabilities to go through the process of photovoice and produce the photovocie storyboards?
2	Community-based inclusive program	Develop a program plan using the program planning model based on the results of needs of assessment.
		Develop an implementation plan for this group of users.
3	Strategies to enhance empowerment and participation	Based on your program design, outline and describe the practical skills in service provision in relation to the domains of health, rehabilitation, social and empowerment as stated in the CBR matrix.

#### Procedures

The first step of the protocol is knowing the scenario and problems. Students are oriented towards problems in their contexts through a short talk or guided visit to a part of the community. They are then instructed to form groups of six students and assigned checkpoint questions based on specific real-world situations. Each group must analyse facts and issues related to their assigned situation and generate a hypothesis to offer a solution. With awareness of knowledge gaps or deficiencies, students are expected to obtain resources from libraries, the internet, and other sources to enhance their knowledge and gain new perspectives and insights to help solve their problems. Consultation with course tutors can be arranged regularly to facilitate learning and the group process. In the reporting session of each checkpoint, each group must summarise what they have learned and give a short presentation on how their newly acquired knowledge is being applied to problem-solving. All knowledge learned and completed work at different checkpoints must be recorded in e-portfolios. Guidance and formative feedback is given by the course tutor in tutorial sessions. Presentation materials and group progress reports are submitted to the course tutor to document each group’s process and progress.

Each checkpoint builds upon the previous one because the process of knowledge acquisition is cumulative. At the end of each semester, each group must show their e-portfolios and give a class presentation to share their learning experiences with other classmates. This final presentation demonstrates the learning outcomes. Presentation content normally includes a comprehensive review of the problem, possible solutions, and practical strategies for action. Students are encouraged to present their outcomes divergently and innovatively; the presentation format should not be limited in words and photos, and should involve the use of new media and multimedia (e.g., video clips to demonstrate how to implement a program). To consolidate learning, each final presentation is followed by an open forum or debate, where students are assumed to have learned sufficient knowledge, theories, and critical thinking skills, and thus are capable of conducting a debate on critical issues by offering insightful opinions. The rationale for each debate or open forum is to exchange knowledge, thereby enriching the knowledge repertoire of everyone involved.

Assessment in PBL should be holistic and performance-based because practical skills developed in PBL cannot be sufficiently assessed through traditional paper-based examination. A coherent learning history and observable outcomes are revealed in each student’s e-portfolio, which is regarded as a learning outcome for assessment. Marking rubrics are based on the relevance of the content, organisation, and framework, as well as supporting evidence, knowledge integration, whether the target problem has been addressed, and the clarity and accuracy of concepts and theories.

### Part II: evaluation of the outcomes and experience of learning

Part II studied the impact of the PBL framework on the learning processes of students. A case study approach was adopted to examine the learning process and outcomes using the PBL protocol. Case study research can include multiple cases with quantitative and qualitative data. The unit of analysis was the student engaged in PBL. The proposition was that PBL can facilitate active learning for students to enhance motivation, knowledge acquisition and application. Evidence was collected from a variety of sources to determine the impact of PBL on learning.

#### Case selection

A total of 133 students in bachelor honours degree programmes at two universities were recruited. Of these students, 86 were enrolled in an occupational therapy program, and the other 47 were enrolled in a special education program. They were recruited from three courses, namely ‘Culturally Relevant Practice’, ‘Sensory and Physical Disabilities’, and ‘Enabling Occupation in Community’. Ninety-two of these 133 students were in their first year of study, and the other 41 were in their third year of study.

#### Data collection

Course tutors conducted the courses according to the developed protocol. For all courses, course content was structured as three modules, with each module presented as a checkpoint to track the learning process. CBR essential components were embedded in the content of these courses. The themes and problem scenarios simulated the commonly concerned issues in the real CBR context. The themes and problem scenarios of the three courses are shown in Table 4. Data on impact of PBL on students’ learning was collected through questionnaires, focus group interviews, and detailed examinations of related documentation such as reflective journals and e-portfolios.

#### Instrumentations

***Focus group interviews*** were conducted to analyse the students’ experiences, reflections, and perceptions of the learning process. All students were invited to join the focus group sessions to ensure the information collected was complete. In the focus group interviews, the students were guided to discuss their opinions of using e-portfolios to summarise and integrate their learning outcomes and PBL compared with more traditional learning methods in learning CBR.

***A questionnaire*** with closed- and open-ended questions was designed and administered at the end of the course to collect data on students’ perceptions on effectiveness of learning. The closed-ended questions comprised 15 items. The questionnaire assessed the following three aspects: (1) perceived self-competency, (2) group effectiveness, and (3) learning and teaching effectiveness.

***An e-portfolio*** is a personalised, web-based collection of work, responses to work, and reflections that is used to demonstrate skills and accomplishments in a variety of contexts over a certain period. Each group was required to submit an e-portfolio to the course instructor at the end of the course. The e-portfolio showed the outcomes of learning, and it allowed the course instructor to assess how well students had acquired and applied knowledge. An e-portfolio is a collection of electronic evidence assembled and managed by students on the internet. Such evidence may include text, files, images, multimedia, blog entries, or hyperlinks. E-portfolios are more flexible than traditional portfolios for constructing a coherent learning narrative [43]. E-portfolios are usually managed and shared using social platforms and social media networks. They offer a stimulating environment because of their audiovisual features. Students can organise their portfolios through various formats and platforms. Most notably, e-portfolios are not constrained by time and location and are therefore well suited to modern learning styles [44]. They are particularly suitable for PBL because they enable students to organise and reflect on knowledge acquired in classes, and this enhances the effectiveness of the method. An e-portfolio can function as a platform for demonstrating the process and learning outcomes of a course [45]. E-portfolios can demonstrate students’ abilities for self-expression, organisation, critical thinking, and reflection. As e-portfolios are managed online, students can maintain them dynamically over time. In addition to the e-portfolio, students were required to keep and submit a group reflective paper that recorded their learning experience in the course and their personal reflections.

### Data analysis

Both quantitative and qualitative analysis was used in this study.

#### Quantitative analysis

Descriptive statistics were used to analyse students’ evaluation of the effectiveness of using PBL pedagogy to learn CBR. Psychometric properties of the questionnaire were checked using SPSS 25.0. Internal consistency and item-total correlation were examined to confirm reliability and validity.

#### Qualitative analysis

All focus group interviews were audio-recorded and transcribed. Content from e-portfolios was extracted and recorded in a data collection template. Thematic analysis was conducted on all qualitative data. The investigator read and reread all data. Emerging themes were systematically recorded with data arranged and collated according to categories. Using replication and pattern-matching logic, within-and across case analysis were conducted to determine the processes and outcomes of using this PBL protocol in teaching CBR.

## Results

***Focus group interviews*** were conducted to examine the students’ perceptions of PBL. A total of 14 focus group interview sessions were held, with each group comprising five or six students. The results of content analysis and thematic mapping are shown in Table 5. The themes emerged on the comparison of PBL to traditional mode in teaching were “impressive”, “enhanced social participation”, “critical” and “realistic and practical”. Compared with more traditional learning methods, PBL is regarded as an effective, realistic, and practical method that enables critical thinking and social participation capabilities to be enhanced. Examples of quotes are shown as below.

*‘I have gained different knowledge and skills in the three stages of the PBL project. For the stage in which we worked on the CBR theories and practical skills, we were required to link what had been learned to our development of a practical activity plan. The process taught us how to transfer knowledge from a theoretical level to a practical level.’*

*‘It inspired me to actively search for reading materials regarding physical disabilities. The checkpoint meetings helped guide us in a right direction as we worked on the rationales of our activity plan. It was very good that we could ask questions and have discussions with the tutor concerning our project. Field visits to the local community helped us to understand physical disability in a real context.’*

*‘There were opportunities for us to apply the concepts of participation and inclusion in our planned programme. We attempted to integrate the idea of inclusion and remove the barriers that restrict people with disabilities from accessing the community. We also learned how we could facilitate the participation of people with disabilities and involve them in various life situations’.*

However, some students reported sometimes losing focus; therefore, guidance from course tutors was required. Examples of quotes are shown as below.

*‘We discovered so much information. It is a bit hard to present many details in a limited time’.*

*‘At the beginning, our group did not have a thorough understanding of the theories. We made quite a lot of mistakes in our work and felt discouraged in the initial stage. After several consultations, we gained a better understanding of how we could apply such theories and were able to propose an appropriate plan to work on the project’.*

The themes emerged regarding the use of e-portfolio were “creative and attractive”, “integrative and comprehensive”, and “practice constraints”. The students liked the use of e-portfolios to summarise learning outcomes because the portfolios were all creative and attractive. Comprehensive recording and integration of the learning process is enabled through the compilation of e-portfolios, which provide a great sense of satisfaction and achievement upon completion of the project. However, the mastery of information technology (IT) skills and other technical aspects were common obstacles encountered by the students. Examples of quotes are shown as below.

*‘Using multimedia and taking videos with my groupmates to show the outcome of our project was fun’.*

*‘It was difficult to learn how to use the software to compile an e-portfolio’.*

The results of the focus group interviews revealed that the students perceived the learning process to be effective because it enables interaction and exchange, enhances engagement in the learning process, and offers inspiration. Students expressed learning was a collective process that involves interaction, mutual sharing, and exchange. In the project, the students supported one another by identifying challenges related to the given scenarios. Learning among students was enhanced through peer observation, idea exchange, and feedback. Examples of quotes are shown as below.

*‘We discussed concepts together and generated ideas. If someone had some misunderstanding of a concept, another groupmate would help to clarify and explain it’.*

*‘Groupmates joined hands together to face problems in the process’.*

*‘We have learned to cooperate and work with different types of groupmates. As in the future, we must be able to cooperate with other professionals or team members. It is important to get everyone involved in the interaction and encourage them to express their ideas and concerns regarding the discussed issue’.*

***Content analysis of the e-portfolios*** of all groups revealed that using e-portfolios could reflect conceptualisation, practicality, and knowledge application (Table 6). Regarding conceptualisation, CBR theories were described and contemplated, sometimes with support from evidence and the development of a conceptual framework to illustrate thoughts and ideas. Practicality was addressed in all groups by covering context-related materials with the use of real cases or examples. Knowledge application was evidenced through group descriptions of the implications on real-life practice. Ethical issues were mentioned by most groups, enabling the students to reflect and gain insight. The presentation style of the e-portfolios was innovative and creative, with frequent use of microfilms, video clips, and animations. Use of diagrammatic presentation aspects such as flow charts and mind maps enhanced the illustration of thoughts and ideas. The students used various platforms to produce and store their e-portfolios. In addition to electronic files for storing information, some of these platforms enable social communication among members to contribute to a final product. Most groups used advanced IT to produce microfilms, video clips, and animations. Technology-based cooperative learning is a gratifying process because it yields an end product. However, most of the groups encountered technical difficulties.

**Table 5:** Content analysis of focus group interview

Themes	Subthemes
The use of e-portfolio	
1. Creative and attractive	Use of multi-media.
	Use of derivative work.
	Colourful presentation.
	Use of animation.
	Interesting and fun.
	With end product that brings high sense of satisfaction.
2. Integrative and comprehensive	Different perspective.
	A mind-mapping process.
	Complete tracking of the learning process.
	High demand on the ability to conceptualize issues.
	Thorough examination of problems.
3. Practical constraints	Time-consuming.
	Technical issues.
	Web-based communication platform.
PBL as compared with traditional mode of teaching and learning	
1. Impressive	Context specific
	First-person sharing.
	Knowledge retained better.
	Application of knowledge.
	Active seeking of information.
	In-depth learning.
	Able to visualize the problems and constraints in real world.
	Consolidation of learning through different stages
2. Enhanced social participation	Fun and interesting.
	Collective learning.
	Interactive and enhanced imagination
	Stimulating.
	Foster good memories.
	With frequent interaction
	Enhanced teamwork and presentation skills
3. Critical	Taking different perspective
	No model answer
	Difficult to get the right focus
	Confront with the real problems
	Inspired with newly generated ideas
4. Realistic and practical	Outreached to the community.
	Site visits.
	Simulated activities in the real context.
	Experiential learning.
	Real.
	Focus on real life application.

**Table 6:** Thematic mapping of content in e-portfolio

	Group												
	1	2	3	4	5	6	7	8	9	10	11	12	13
Conceptualization													
Theories embedded	X	X	X	X	X	X	X	X		X	X		
Support with literature	X	X	X	X	X	X	X		X		X	X	
Development of conceptual framework		X	X	X	X						X		
Practicality													
Context-related materials	X	X	X	X	X	X	X	X	X	X	X	X	X
Cultural values embedded	X	X	X	X	X	X	X	X		X			
Use of real case studies / examples	X	X			X		X	X	X	X	X	X	X
Application													
Included implications on practice	X	X	X	X	X	X	X	X	X	X	X	X	X
Ethics embedded	X	X	X			X	X		X	X		X	
Reflection	X	X	X	X	X	X	X	X	X	X	X	X	X
Presentation format													
Animation and video clips	X			X	X				X	X	X	X	X
Diagrammatic presentation and mind map	X	X	X	X	X	X	X		X		X		X

***A questionnaire*** was conducted for triangulation. Psychometric properties of the questionnaire were examined. The Cronbach’s alpha value, representing the internal consistency of the questionnaire was, 0.916, the Cronbach’s alpha if an item was deleted for all items was greater than 0.9, and the inter-item correlation of all items was greater than 0.3 [46], The reliability of the questionnaire was therefore confirmed. The results of the questionnaire (*n* = 133) demonstrated that PBL was perceived by students as an effective teaching and learning method (Table 7) that enhanced CBR competence and promoted student-to-student interaction and exchange. As summarised by responses to the open-ended questions, the students were satisfied with their acquired deeper understanding of knowledge that resulted from the learning process. The students were actively engaged in the learning process and the process was perceived to be inspirational. PBL was able to address student needs in the learning process. However, a few students suggested that the tutor should have taught more about related skills and knowledge rather than allotting large amounts of time for consultation and facilitation.

**Table 7:** Students’ perceived effectiveness of PBL

	Mean score	Standard deviation
CBR competence		
Understanding the needs of service users	3.30	0.51
Comprehending CBR theories and concepts	3.36	0.57
Management practical strategies	3.17	0.60
Group work effectiveness		
Satisfaction with group performance	3.30	0.62
Positive atmosphere and teamwork	3.51	0.55
Teaching and learning		
Facilitating Interaction and exchange	3.28	0.54
Engaging	3.23	0.48
Inspiration	3.34	0.52
Addressing students’ need	3.15	0.55
Achieved learning effectiveness	3.23	0.56
Critical thinking	3.34	0.52
Enhanced organization of content	3.21	0.59
Enhanced feedback	3.23	0.63
Achieved learning outcomes	3.28	0.50
Enhanced knowledge and skills acquisition	3.29	0.58

No. of participants = 113

The highest score for each items is 4

4 = strongly agree

3 = agree

2 = disagree

1 = strongly disagree

### Converging all evidence

The data from all of the sources were integrated to reveal the impact of PBL on students’ learning. The synthesis showed that PBL affected students’ perceptions of the learning process, problem-solving, motivation and satisfaction with learning. They described the learning process as impressive, realistic, practical and effective. The use of multimedia was regarded as attractive and creative. The group work process was appreciated, because it enhanced social participation and enabled interaction and exchange. However, the students also thought that the PBL process was complicated and they sometimes lost focus. They considered guidance from tutors to be essential. Some of the students found the use of technology challenging. Their problem-solving abilities and learning outcomes were reflected in their e-portfolios. In addition to revealing the learning outcomes, their work reflected their ability to conceptualise, organise and do practical tasks. Their ability to apply knowledge to solve problems, make ethical choices and self-reflect were also demonstrated. The questionnaire revealed the perceived effectiveness, motivation and satisfaction with the learning process. The process was regarded as effective in enhancing their perception of CBR competence. Most of the participants reported that their comprehension of CBR theories and concepts was enhanced. They were satisfied with the learning process because it was inspirational. It helped to boost their ability to understand the needs of people with disabilities.

## Discussion

### Comparing to literature

This study developed and tested a PBL framework and protocol to teach CBR in higher education. The central steps of PBL as stated in this framework (Fig. 1) are (1) knowing the problem scenario, (2) identifying facts, (3) identifying knowledge gap, (4) develop resource, (5) gaining insights to apply knowledge, and (6) reflecting on knowledge gain. The Maastricht seven-step model of PBL has been criticized as overemphasizing on problem solving rather than learning because it adheres to a hypothetico-deductive approach [47]. Other approaches may put greater emphasis on identifying and filling of knowledge gaps [39]. Other than choosing just one or two models [30, 31], this study has developed, built and tested a framework based on a synthesis of evidence. The framework and protocol as developed in this study emphasize on learning and they were built upon an integration and synthesis of evidence. As in the literature, the approach of PBL were widely interpreted and practice varied in different context [48]. PBL is mostly presented in literature as a set of concepts with gross procedures [33, 38]. Concrete guidelines and practical procedures to guide practice were lacking. This study adds a practical way to incorporate PBL in teaching.

Learning is cumulative and new learning depends on what has been learned previously [49]. Other than solely describing the steps involved in the PBL process [29, 32], our framework states also the three important phase of learning: problem analysis phase, self-directed learning period and reporting phase. It supports that the nature of learning in PBL is sequential: start from problem analysis phase, to self-directed learning, and to the reporting phase [49]. Knowledge acquisition can be progressive and learning of clinical skills usually starts from a discursive and theoretical level and then progress to an active and practical level. Schmidt et al. [49] proposed two hypotheses to explain how learning is driven in PBL: an activation-elaboration hypothesis and a situational interest hypothesis. These hypotheses support our framework. “Knowing the problem scenario” in a small group leads to the activation of prior knowledge; “identifying facts and knowledge gap” drive learning by generating situational interest; and “develop resources, apply knowledge and reflection” provides opportunities for elaboration on that knowledge. Both group collaboration and individual knowledge acquisition contributes equally to learning in PBL.

This study has contributed to the evidence base of CBR. This is the first study that systematically examine the use of PBL in teaching CBR. Although CBR has been implemented in over 100 countries and evolving for over three decades [50], the evidence base for an effective pedagogy to teach CBR has long been lacking [51]. This study presented the building of a framework based on the review of PBL literature and an extensive qualitative study of 133 students in actual settings. This framework has the potential to inform educators, practitioners and researchers about the components of an effective PBL pedagogy to teach CBR.

### Effective course design that incorporates PBL

This PBL framework and protocol might help course instructor to develop effective course designs. First, the essential procedures of PBL were identified as follows: identifying facts, identifying knowledge gaps, developing resources, gaining insights into applying knowledge and reflecting on knowledge gains. These steps enabled students to assume active roles in organising conceptual ideas and linking these ideas to theories. Students were guided to adopt self-directed learning. That is, they were encouraged to seek resources from a variety of sources to generate and test hypotheses to solve real-life problems. This process enabled the students to actively seek practical methods to solve problems and figure out dilemmas in real settings. Higher levels of clinical reasoning and critical thinking were achieved through this process. Second, the content of a course can be arranged into progressive submodules (or checkpoints), so that the students can consolidate the knowledge they gained in the previous checkpoint before continuing. The PBL protocol developed in this study offers a practical and concrete guide for course instructors to lead the students through the learning process. Learning is a cumulative process that emphasises the iterative identification of facts and knowledge gaps, resource development, and consolidation through knowledge application and reflection. Third, the study showed that small group tutoring facilitated learning. The students perceived the learning process to be effective, because it enabled interaction and exchange, enhanced engagement in the learning process and offered inspiration. Learning among students was enhanced by peer observation, idea exchanges and feedback. Fourth, the study showed that learning can be real and community-based. This study demonstrated that real learning can occur beyond the classroom boundaries and in the local community. In this study, the students were encouraged to reach out to the local community to gather information, conduct on-site visits, and conduct experiential learning activities in real-life environments. The students were able to visualise clear links between what they were learning and what they were attempting to achieve in practice. The PBL curriculum in this study was designed around health care scenarios inspired by real-life situations and social dilemmas. The learners were enabled to sharpen their clinical reasoning, decision–making, and case conceptualisation skills as they worked through each checkpoint and scenario. Sound rationale, logical justification, and practical solutions are essential in working through problem-based scenarios in real-life situations. Fifth, PBL requires an effective tool to report, document, and reflect the process and outcomes of learning. The use of e-portfolios was found to be effective in documenting learning processes and outcomes in all three courses. Implications on practice should be explained with consideration of ethical practices, and reflection during the process is essential in demonstrating that the learning experience was well-integrated and consolidated.

However, lecturers or course instructors must also acknowledge the limitations of the PBL approach. Some students reported difficulty in using the technology needed to formulate their e-portfolios. Thus, a good infrastructure that provides sufficient technical support to students is required. The institution is required to be well-prepared and equipped in terms of teaching and learning support provided to students, including library resources, hardware, and IT support. Furthermore, many students reported losing focus during the process of learning. In this study, the students sometimes expressed confusion as a consequence of losing focus during the inquiry process. As PBL emphasises self-directed learning, not all essential theories for particular subjects are specified. When PBL is implemented, students may overlook essential information because of the unspecific focus of learning in PBL. It is possible that an inexperienced learner might overlook the information that seems irrelevant to the theme of the course. Students might also spend excessive amounts of time learning theories and knowledge, resulting in target abilities such as problem-solving skills not being acquired to a sufficient degree. Because the instructional and tutorial styles in PBL are different from those of traditional didactic teaching methods, both the students and instructors must be well oriented and prepared for the new mode of learning. Students must develop the responsibility of owning the learning process and realise that a certain amount of time is required for discussion in PBL. Therefore, educators must be trained to skilfully and sensitively give feedback to facilitate the students in the learning process.

### Community-based rehabilitation

The PBL pedagogy is found to be an effective method for teaching CBR because it enables the cultivation of the appropriate mindset, attitudes, knowledge, and skills of a CBR practitioner. The results of this study indicated that this approach helped students to practically examine the context-related knowledge and transform what they had learned into implications for CBR practice. In the e-portfolios, the majority of the groups correctly conceptualised the CBR theories, found good support in the literature and used real case studies or local examples to illustrate how they solved the problems. When triangulated with the results obtained from the questionnaire, the results showed that the participants thought that the PBL pedagogy enhanced their comprehension of CBR theories and concepts, their understanding of the needs of the service users and their management of CBR practical strategies. CBR practitioners must work in teams and with various counterparts in the community. It is essential for CBR practitioners to be able to work effectively as part of a team and with organizations with a diverse background in the local community. Educators must provide learning experiences that establish knowledge-seeking behaviours in future CBR practitioners. Reasoning in a CBR context requires recognising and understanding basic human conditions, a thorough comprehension of the person-centred approach, an awareness of all related CBR contextual factors, and knowledge regarding the application of CBR strategies in a dynamic context [52]. Empowerment of individuals and the community is an essential component of a CBR program. Strategic thinking and problem-solving skills are required to build the capacity of a community. The PBL model enables the teaching of CBR to be flexible, stimulating, and practical. Using this model, theories and concepts as well as practical strategies and tools can be taught in such a manner that students come to comprehend both knowledge and skills in case management, problem solving, and community development tasks [8].

## Conclusion

This study developed and tested a PBL protocol for use in the education of CBR-related courses in higher education. PBL is regarded as a cumulative and iterative process for gaining insights into problem-solving in real-life situations. By applying the PBL protocol, the students in this study were guided through the phases of problem analysis, self-directed learning, and reporting. Upon completion of the learning process embedded in the course, both theoretical and practical levels of knowledge acquisition had been achieved. The students asserted that the PBL protocol had enhanced their motivation and learning quality. Special consideration of the preparation of the institution, instructors, and students is required to ensure the success of PBL.

## Data Availability

The data contributing to these analyses are held on a secure database and all data generated or analysed during this study are not publicly available as this may be linked to specific program and staff. Sharing of such data may breach confidentiality.

## Abbreviations

CBR: Community-based rehabilitation
PBL: Problem-based learning
